# Qualitative hypotheses

**DOI:** 10.12688/openreseurope.19736.1

**Published:** 2025-03-07

**Authors:** Veli-Matti Karhulahti

**Affiliations:** 1 University of Jyväskylä, Jyväskylä, Central Finland, Finland

**Keywords:** Epistemology, methodology, preregistration, registered reports, meta-science

## Abstract

Hypotheses are not commonly disclosed in qualitative research. This can be largely attributed to many qualitative methods not being optimal or suitable for hypothesis testing, and the epistemological curiosity in qualitative research often lacking interest in testing. Nonetheless, qualitative researchers, too, do often operate with hypotheses even though they do not test them. In this essay, I discuss qualitative hypotheses (QHs) as a means to disclose hypotheses without testing them. In particular, the usage, functions, and limitations of QHs are proposed with a contextualising historical overview.

## Plain language summary

This concise essay is an encyclopaedic entry on qualitative hypotheses (QHs), which are conceptual tools for researchers to disclose their expectations without testing them. As such, the essay provides an efficient outline of QHs and aims to serve as a practical source that can help researchers decide whether using QHs is beneficial for them or not.

## Introduction

This essay provides a concise meta-methodological overview of *qualitative hypotheses* (QHs), which have recently emerged in the literature along with the proliferation of qualitative preregistration (e.g., Haven *et al.*, 2020) and registered reports (e.g., Demkowicz & Hickman Dunne, 2024). QHs were introduced to scientific practice some years ago ‘accidentally’ and remain epistemologically as well as pragmatically undiscussed. The goal of this essay is to initiate that discussion and help researchers assess whether QHs might be useful for them or not.

## Conceptual background

Etymologically, the term ‘hypothesis’ derives from Greek, merging the prefix *hypo* (below) with *thesis* (statement). *Oxford English Dictionary* gives hypothesis multiple active meanings, such as the following.

*(b)*  *A supposition or conjecture put forth to account for known facts; esp. in the sciences, a provisional supposition from which to draw conclusions that shall be in accordance with known facts, and which serves as a starting-point for further investigation by which it may be proved or disproved and the true theory arrived at*

When practicing science ^1^, we operate with hypotheses each and every moment. Following this, conversations in the philosophy of science sometimes summon the term ‘auxiliary hypothesis’ to account for the idea that our underlying assumptions, too, belong to the realm of hypotheses (e.g., Lakatos, 1978). In this sense, hypotheses are not much different from the assumptions that make our work possible.

For a long time, the above state of affairs has been confused by a distinct epistemic activity, ‘hypothesis testing’, and statistical hypothesis testing in particular (e.g., Field, 2022). The term ‘hypothesis’ carries specific meanings in such contexts, and is often further complicated by derivative notions such as ‘descriptive hypothesis’, ‘formalised hypothesis’, and ‘statistical hypothesis’. I leave the above terms to be debated by hypothesis-testers and discuss QHs as a tool outside test frameworks. The purpose of a QH is simply to disclose what researchers expect to find in a study that they are about to carry out. I start with a historical note and continue with meta-methodological scrutiny.

## Historical background

One reason for me to write this essay is that I remain guilty of having co-introduced QHs to the scientific discourse some years ago. Back then, the concept was not presented as an epistemological or meta-methodological contribution, but conceived for pragmatic reasons to meet the technical expectations of a *registered report* plan (for readers unfamiliar with registered reports, see e.g., Henderson & Chambers, 2022).

Apparently, our study was the first registered report driven by qualitative data and methods, which put us into a position of needing to creatively navigate submission guidelines that were generally intended for hypothesis testing designs. When having our phenomenologically oriented research plan in peer review—before the study was carried out—we followed the guidelines and inserted two hypotheses. Alas, as our work did not aim to *test* any hypothesis, we provided the following description:

“By qualitative hypotheses we mean that the goal is not to seek confirming and falsifying evidence, and we will not claim the data to (not) support the alternative hypotheses (or null). This is because qualitative research, at least in the presently applied interpretive phenomenological form, is not well suited for null hypothesis significance testing. That said, we do consider phenomenological research suitable for another kind of significance testing—significance as *meaning*—and because these forms of significance already have an existing basis in the literature, disclosing this basis openly in the form of qualitative hypotheses will add to the transparency of the work. Accordingly, we consider it important to set hypotheses *not to test hypotheses, but rather because we want to disclose our hypothetical biases*.” ( Karhulahti *et al.*, 2022; future tense has been changed in final publication)

Reading the above now years later, I would rephrase some of the terms, but in general, the message holds. When doing qualitative research—or any other research for that matter—we usually have expectations about what we are going to find out. We may not be fully aware of those expectations, as they are likely to align with our fundamental beliefs and positions, which often entail reflexive effort to be made visible. We may also operate under disciplinary, theoretical, or ‘state of art’ understandings of our fields, which shape what we (are expecting to) see in the results. Reflecting on these aspects was the intended purpose of our QHs. A year later, in a primer for qualitative registered reports, we wrote another section that aimed to summarise this idea:

“It is uncommon (but not impossible) for qualitative research designs to test hypotheses. On the other hand, it is also possible to set hypotheses without testing them. These ‘qualitative hypotheses’ (QHs) can be used in a similar way as positionality statements, i.e. to report the team’s prior beliefs and hypothetical biases, which can affect the study design and its procedures… Unlike positionality statements, QHs are based on previous data, literature, and theory, which have influenced the study design and may influence data interpretation, thus being akin to ‘priors’ in Bayesian statistical designs” ( Karhulahti *et al.*, 2023)

Beyond the above two quoted paragraphs, conceptualisations of QHs have not, to my knowledge, entered the meta-methodological discourse. Thus, I will briefly elaborate on the concept by addressing three core issues: usage, functions, and limitations.

## Usage, functions, and limitations

**Usage**. By definition, any hypothesis is a prognostic concept. The utility of QHs—not unlike testable hypotheses—is based on having them *before* the study is carried out. Adding QHs after a study is completed would not be useful. Researchers who have completed a study may wish to reflect on their updated priors or shifted perspectives, but such reflections should be kept distinct from QHs to avoid conceptual confusion. QHs serve primary as components of qualitative preregistrations and registered reports, i.e. formats that involve a pre-analytic phase of reflection.

As it goes with other scientific hypotheses, QHs can be used with different methods and types of data. The word ‘qualitative’ carries a double meaning in QHs, referring to both the descriptive nature of QH formulation and its plausible application in qualitative research in particular. Unlike statistical priors, QHs are qualitative descriptions that do not aim to formalise an expectation. The accuracy and depth of a QH depends on the researchers and their study design, and such features should be decided with pragmatics in mind. As to the plausible context of application, in turn, QHs tend to match best with qualitative research traditions where other types of hypotheses are rarely used. Naturally, it is possible to include QHs in quantitative and other research designs too (see Smith, 2005), but that should be done knowingly, i.e. with an identified function and relevance.

**Functions**. At the time of writing this, I see three potential functions associated with QHs. First, adding QHs either to a preregistration or a registered report can encourage researchers to *reflect* on their expectations. Similar benefits have been proposed for preregistration and registered reports in general, as they encourage—and in the latter case also potentially enforce—researchers to think through various details of the research plan before conducting the work. Haven & van Grootel (2019) phrase this clearly:

“Preregistration does not have to challenge the subjectivity crucial to qualitative research; on the contrary, it underlines the importance of subjectivity by encouraging qualitative researchers to reflect upon their *a priori* values by enabling the researcher to make these values transparent for other researchers from the start of the research” (p. 237)

Consequently, and second, adding QHs to a preregistration or a registered report also contributes to *transparency*. When writing down one’s expectations as a QH, a researcher goes through a reflective process where they mirror subjective pre-knowledge of the question under study. The depth and fidelity of such process is researcher-specific, but if and when disclosed, also subject to external evaluation and critical discussion. Regardless of whether the researchers’ expectations are met or not, having them spelled out in a QH will enable readers, reviewers, and the researchers themselves look back. Obtaining findings that go against QHs should not have negative impact on the evaluation of the work, but it merely implies that something unexpected has been discovered. Verbal descriptions of QHs should not be optimised to be ‘supported’ later (e.g. by vague language that covers a maximal range of outcomes); instead, they should disclose relevant beliefs, which are valuable to reflect on later.

Third, as adding QHs to a preregistration or a registered report documents the researchers’ expectations as metadata, this allows both readers and researchers to *update* their beliefs in a dialogic manner. Because QHs are not tested, it may not always be useful to discuss them in detail after the study has been carried out. However, when the findings go against or otherwise differ from registered QHs, this creates a transparent opportunity to address such issues and reflect on how and why the process has (not) updated the researchers’ views regarding the studied topic.

**Limitations**. It is important to stress that QHs do not automatically improve a study and they should not be applied without considering how they contribute to a specific study. For example, it is possible that researchers do not have any meaningful expectations about their findings, which may be a sufficient reason to omit QHs. On the other hand, because one of the functions of QHs is to explicitly encourage reflecting on one’s expectations, it is important to distinguish a lack of expectations from a lack of reflection. We should be cognisant and remain open to other (potentially numerous) conditions under which QHs may not be useful; for instance, in large studies with multiple authors it may be impossible to utilise QHs in a pragmatic manner.

Finally, the concept of QHs is not mine to own, and how I have visioned them to operate remains to be challenged, ignored, and refuted. Those willing to use QHs in any modified form that suits their needs best are encouraged to do so. In short, researchers should contemplate case-by-case whether the above or other functions of QHs yield their study meaningful benefits. The following footnote documents two existing examples. ^2^

## Ethics and consent

Ethical approval and consent were not required.

## Data Availability

No data associated with this article.
