# Qualitative hypotheses

**DOI:** 10.12688/openreseurope.19736.2

**Published:** 2025-08-08

**Authors:** Veli-Matti Karhulahti

**Affiliations:** 1 University of Jyväskylä, Jyväskylä, Central Finland, Finland

**Keywords:** Epistemology, methodology, preregistration, registered reports, meta-science

## Abstract

Hypotheses are not common in qualitative research. This can be largely attributed to many qualitative methods not being optimal or suitable for hypothesis-testing, and the epistemological curiosity in qualitative research often lacking interest in testing. Nonetheless, qualitative researchers, too, do often enter research projects with expectations, which are similar to hypotheses. These expectations can be useful to disclose and reflect on before a study is carried out. In this essay, I discuss qualitative hypotheses (QHs) as a means to disclose such expectations without testing them. In particular, QHs are presented as a tool that can be useful with recently proliferated reporting formats, such as a preregistration and registered reports, which involve a pre-analytic phase of written reflection.

## Plain language summary

This concise essay is an encyclopaedic entry on qualitative hypotheses (QHs), which are tools for researchers to disclose their expectations without testing them. As such, the essay provides an efficient outline of QHs and aims to serve as a practical source that can help researchers decide whether using QHs is beneficial for them or not.

## Introduction

This essay provides an encyclopaedic meta-methodological overview of *qualitative hypotheses* (QHs), which have recently emerged in the literature along with the proliferation of qualitative preregistration (e.g., Haven *et al.*, 2020) and registered reports (e.g., Demkowicz & Hickman Dunne, 2024). As QHs remain epistemologically and pragmatically undiscussed, this essay aims to initiate such discussion and help researchers assess whether QHs might be useful for them or not.

## Conceptual background

Etymologically, the term ‘hypothesis’ derives from Greek, merging the prefix *hypo* (below) with *thesis* (statement). *Oxford English Dictionary* gives hypothesis multiple active meanings, such as the following.

“A supposition or conjecture put forth to account for known facts; esp. in the sciences, a provisional supposition from which to draw conclusions that shall be in accordance with known facts, and which serves as a starting-point for further investigation by which it may be proved or disproved and the true theory arrived at”

The above description is easily confused by a distinct epistemic activity, ‘hypothesis-testing’, and statistical hypothesis-testing in particular (e.g., Field, 2022). The term ‘hypothesis’ carries specific meanings in such quantitative contexts and is rarely used in qualitative research designs (e.g., Dehalwar & Sharma, 2024). This conceptual domain is often further complicated by derivative notions such as ‘descriptive hypothesis’ and ‘formalised hypothesis’. I leave those terms to be debated by hypothesis-testers, as in the context of QHs, ‘hypotheses’ refer to provisional expectations that are not tested.

When practicing science ^1^, we operate with provisional expectations and suppositions each and every moment. Nevertheless, ‘hypothesis’ is but one of many terms, which have been used to address researchers’ suppositions and expectations. For example, Maxwell’s (2008) approach to qualitative research utilises the term ‘conceptual framework’. Maxwell explicitly distinguishes the conceptual framework from the literature review, as the former also includes

“communication with other researchers, and your own experience and pilot studies [as well as] theories that are particularly relevant to your research… your purpose is not only descriptive, but also critical; you need to treat ‘the literature’ not as an authority to be deferred to, but as a useful but fallible source of ideas [i.e.] the conceptual framework for your research study is something that is constructed, not found. It incorporates pieces that are borrowed from elsewhere, but the structure, the overall coherence, is something that you build, not something that exists ready-made” (p. 223)

In a similar but not identical way, the purpose of a QH is to disclose what researchers expect to find in a study that they are about to carry out. In contrast to the conceptual framework, however, the QH is not a holistic frame, but an articulation of the most relevant expectations regarding the upcoming findings before initiating work.

Because the expectations of a QH are not to be tested—as in hypothesis-testing paradigms—it can be useful to conceive of QHs as ‘scientific hypotheses’ in a metaphorical sense (e.g., Blumenberg, 1979; Goodman, 1968; Johnson, 2007). Namely, the QH is a tool with bundled features, many (but not all of) which have previously been associated with scientific hypotheses and testing. Although the QH carries features of scientific hypotheses, it does so essentially as an adaptation to the technical evolution of science, which has recently produced various pre-study reporting practices. The QH is not a novel concept for the research process itself but a tool for pre-study reporting, which did not exist in the past.

## Historical background

One reason for me to write this essay is that I remain guilty of having co-introduced QHs to the scientific discourse some years ago. Beyond the below two quoted paragraphs, conceptualisations of QHs have not, to my knowledge, entered the meta-methodological discourse. Initially, we did not present the QH as a meta-methodological tool, but conceived it for pragmatic reasons to meet the technical expectations of a *registered report* plan (for readers unfamiliar with registered reports, see e.g., Henderson & Chambers, 2022).

Apparently, our study was the first registered report driven by qualitative data and methods, which put us into a position of needing to creatively navigate submission guidelines that were intended for hypothesis-testing designs. When having our phenomenologically oriented research plan in peer review—before the study was carried out—we followed the guidelines and inserted two hypotheses. Alas, as our work did not aim to *test* hypotheses, we provided the following description:

“By qualitative hypotheses we mean that the goal is not to seek confirming and falsifying evidence, and we will not claim the data to (not) support the alternative hypotheses (or null). This is because qualitative research, at least in the presently applied interpretive phenomenological form, is not well suited for null hypothesis significance testing. That said, we do consider phenomenological research suitable for another kind of significance testing—significance as *meaning*—and because these forms of significance already have an existing basis in the literature, disclosing this basis openly in the form of qualitative hypotheses will add to the transparency of the work. Accordingly, we consider it important to set hypotheses *not to test hypotheses, but rather because we want to disclose our hypothetical biases*.” ( Karhulahti *et al.*, 2022; future tense has been changed in final publication)

Reading the above years later, I would now add that researchers’ expectations never derive solely from the literature but various sources in our subjective histories, and the QH is thus not to be confused with the literature or its review alone (recall Maxwell earlier). Moreover, I would remove the emphasis on ‘hypothetical biases’—even though they remain relevant for some studies—and replace that with a more epistemically flexible ‘researcher subjectivity’ (e.g., Braun & Clarke, 2023). Not all research epistemologies operate with bias, and researcher subjectivity better accounts for those epistemological frameworks as well. 

When doing qualitative research, or any other research for that matter, we usually have expectations about what we are going to find out. We may not be fully aware of those expectations, as they are likely to align with our fundamental beliefs and positions, which often entail reflexive effort to be made visible. We may also operate under disciplinary, theoretical, or ‘state of art’ understandings of our fields, which shape what we (are expecting to) see in the results. Reflecting on these aspects was the intended purpose of our QHs. A year later, in a primer for qualitative registered reports, we wrote another section that aimed to summarise this idea:

“It is uncommon (but not impossible) for qualitative research designs to test hypotheses. On the other hand, it is also possible to set hypotheses without testing them. These ‘qualitative hypotheses’ (QHs) can be used in a similar way as positionality statements, i.e. to report the team’s prior beliefs and hypothetical biases, which can affect the study design and its procedures… Unlike positionality statements, QHs are based on previous data, literature, and theory, which have influenced the study design and may influence data interpretation, thus being akin to ‘priors’ in Bayesian statistical designs” ( Karhulahti *et al.*, 2023)

Unlike positionality statements, which disclose the researcher’s general position relative to a research topic (e.g. Penders *et al.*, 2025), the QH is a disclosure of priors explicitly about the *research question* at hand. For example, personal values that are commonly disclosed in positionality statements would be meaningless to fit in most QHs—even though personal values may meaningfully *affect* one’s QHs. Put another way, one and the same statement of positionality could be used for multiple studies of a research project, but each of the project’s studies with distinct research questions would also entail distinct QHs. Next, I elaborate on QHs by addressing their use and functions.

## Use and functions

**Use**. In many qualitative and related research traditions, such as ethnography ( Hammersley & Atkinson, 2019) and grounded theory ( Glaser & Strauss, 1967), scholars standardly reflect on their updated priors or shifted perspectives during and after the work. Such reflections are a vital part of knowledge development; nonetheless, explicitly stating reflexive expectations prior to initiating work sets QHs apart from conventional reflexive practice. Any hypothesis is a prognostic by definition, and accordingly, the utility of QHs is based on having them *before* the study is carried out. Adding QHs after a study is completed would not be useful. Hence, QHs serve primary as tools for qualitative preregistrations and registered reports, which involve written pre-study reflection.

As it goes with other scientific hypotheses, QHs can be used with different methods and types of data. The word ‘qualitative’ carries a double meaning in QHs, referring to both the descriptive nature of QH formulation and its plausible application in qualitative research in particular. Unlike statistical priors, QHs are qualitative descriptions that do not aim to formalise an expectation. The accuracy and depth of a QH depends on the researchers and their study design, and such features should be decided with pragmatics in mind. As to the plausible context of application, in turn, QHs match best with qualitative research traditions where other types of hypotheses are rarely used. Naturally, it is possible to include QHs in quantitative and other research designs too (see Smith, 2005), but that should be done knowingly, i.e. with an identified function and relevance.

**Functions**. At the time of writing, I see three potential functions associated with QHs. First, adding QHs either to a preregistration or a registered report can encourage researchers to *reflect* on their expectations. Similar benefits have been proposed for preregistration and registered reports in general, as they encourage—and in the latter case also potentially enforce—researchers to think through various details of the research plan before conducting the work. Haven & van Grootel (2019) phrase this clearly:

“Preregistration does not have to challenge the subjectivity crucial to qualitative research; on the contrary, it underlines the importance of subjectivity by encouraging qualitative researchers to reflect upon their *a priori* values by enabling the researcher to make these values transparent for other researchers from the start of the research” (p. 237)

Consequently, and second, adding QHs to a preregistration or a registered report also contributes to *transparency*. When writing down one’s expectations as a QH, a researcher goes through a reflective process where they mirror subjective pre-knowledge of the question under study. The depth and fidelity of such process is researcher-specific, but if and when disclosed, also subject to external evaluation and critical discussion. Regardless of whether the researchers’ expectations are met or not, having them spelled out in a QH will enable readers, reviewers, and the researchers themselves look back. Obtaining findings that go against QHs should not have negative impact on the evaluation of the work, but merely implies that the unexpected has been discovered. Verbal descriptions of QHs should not be optimised to be ‘supported’ later (e.g. by language that covers a maximal range of outcomes); instead, they should disclose relevant expectations, which are valuable to reflect on later.

Third, as adding QHs to a preregistration or a registered report documents the researchers’ expectations as metadata, this allows both readers and researchers to *update* their beliefs in a dialogic manner. Because QHs are not tested, it may not always be useful to discuss them in detail after the study has been carried out. However, when the findings go against or otherwise differ from registered QHs, this creates a transparent opportunity to address such issues and reflect on how and why the process has (not) updated the researchers’ views regarding the studied topic.

## Case example

To provide an illustration in practice, I refer to a recent qualitative study ( Hickman Dunne *et al.*, 2025), which investigated how motivations in adolescent social media use relate to mental health (RQ1) and how the experiences of such use manifest from a mental health point of view (RQ2). Utilizing reflexive thematic analysis ( Braun & Clarke, 2023), the authors analysed adolescent (n=26) focus group data and set two QHs before the start the work:

*QH1: We expect heterogeneity in the motivations and experiences of social media use and types of platforms used, especially between different age groups.*
*QH2: We expect that social media experience will be multidimensional with key dimensions like cyberbullying, social comparison, fear of missing out, and social support and connection to be discussed.*

In the current case, the QHs reflected both the authors’ interpretation of relevant literature as well as their subjective beliefs and perspectives on the research questions. Although the study did not involve a pilot, the QHs had been co-produced by three young researchers who had been recruited from a consultancy organisation to enrich the team’s understanding of the topic. After the completion of the study, the authors reflected on their QHs:

“extending our understanding of H1, not only is there between-person variation, including some possible differences across age and gender, but there also appears to be within-person variation. Young people described different and often polarised experiences resulting from similar activities, based on, for example, the context within which they were using social media, their mood state, or an anticipated or actual social interaction… multiple dimensions of social media experience (H2) reflected in the current sample were expressed in a complex way. Whilst we designed the focus group schedule to focus one at a time on each of perceptions, motivations, activities, and experiences, discussion of these was overlapping, indicating that for young people, social media experience is not clearly disentangled along such lines. The learnings relating to the study hypotheses show that the relationship between motivations, behaviours and experiences in relation to social media use may therefore not be as straightforward” (pp. 15–16)

The post-study reflections illustrate the researchers’ updated priors and how their thinking of the subject matter changed throughout the research process. Even though similar reflections would be possible to disclose without written QHs as well, pre-study expectations and beliefs like the above would have been more difficult to recall without having spelled them out as QHs earlier. In other words, the QHs served as a technical tool to support reflexive practice. ^2^

## Limitations

Because using QHs entails technical means to state them before a study is carried out, their application is limited to researchers who have access to (and find their work benefitting from) preregistration, registered reports, or other reporting innovations with a pre-study phase. As tools that support reflexive practice, QHs do not appear to be constrained so much by epistemological differences as they are by technical limits.

It is important to stress that QHs do not automatically improve a study and they should not be applied without considering how they contribute to a specific study. For example, it is possible that researchers do not have any meaningful expectations about their findings, which may be a sufficient reason to omit QHs. On the other hand, because one of the functions of QHs is to explicitly encourage reflection, it is important to distinguish a genuine lack of expectations from a mere lack of reflection.

We should also be cognisant and remain open to other conditions under which QHs may not be useful. For instance, in large studies with multiple authors it may be difficult to utilise QHs in a pragmatic manner. On the other hand, just like ‘collective positionality’ has emerged as a distinct type of reflexivity statement that reflects on team compositions rather than researcher subjectivities (e.g., Penders *et al.*, 2025), it is possible that similar functional variation emerges in the application of QHs in the future.

The concept of QHs is not mine to own, and how I have visioned them to operate remains to be challenged, ignored, and refuted. Those willing to use QHs in any modified form that suits their needs best are encouraged to do so. In short, researchers should contemplate case-by-case whether the above or other functions of QHs yield their study meaningful benefits.

## Ethics and consent

Ethical approval and consent were not required.

## Data Availability

No data associated with this article.
