# Supplementary material for: Qualitative hypotheses
Source: Open Res Eur. 2025 Aug 8;5:64. Originally published 2025 Mar 7. [Version 2] doi: 10.12688/openreseurope.19736.2 (PMC12395124; doi:10.12688/openreseurope.19736.2)
Supplement: Peer review history

# Reviewer report: Reviewer response for version 2

**DOI:** 10.21956/openreseurope.22856.r58123

**Published:** 2025-09-05

**Authors:** Stefan Reichmann

**Affiliations:** 1 Graz University of Technology, Graz, Austria

This is a timely contribution in that it seeks to elucidate the relationship between open research methods, most notably preregistration, and qualitative research (or rather, certain aspects of qualitative research). This is achieved by investigating the role of hypotheses in qualitative research (referred to as qualitative hypotheses).

 My main criticism of the paper is this: Given that it seeks to elucidate the role of hypotheses in qualitative research, I would expect a thorough engagement with qualitative methodologies, specifically as the contribution contains a section labelled “Historical Background”. This suggests that while the author is a qualitative researcher (or seems to be by their own account), the paper is actually not written from the point of view of qualitative research, but rather tries to accommodate a rather incomplete picture of working qualitatively with registered reports (which is a tool specific to a rather narrow understanding of research).

 In particular, qualitative research needs to be thoroughly demarcated from positive science in that each follows very different methodological assumptions. While positive science is characterized by the separation of subject and object, wanting to achieve reliability, reproducibility, and representativeness. This, it tries to achieve through excluding reactivity, e.g. by constructing standardized methods of data collection, and by analyzing data according to statistical models.

 Qualitative research is characterized by intervention (i.e., qualitative researchers are in close contact with their field, they often intervene into their subjects lived experience), process, structuring, and theory reconstruction. In this, qualitative research tries to mobilize the contextual effects between interviewer, respondent, field, and situation, instead of trying to eliminate them (this is, after all, the point of standardized research). Qualitative methods are not standardized by definition (which means, indeed, that “methods” might be a bit of stretch). Contextual effects in positive science correspond to the power effects of qualitative research (domination, silencing, objectification, and normalization). I would like to stress that both models of science are necessary, and both have their strengths and weaknesses.

 However, the paper evidently tries to analyze (or even colonize) qualitative research from the vantage point of positive science, without any deeper engagement with the methodological assumptions of either. Indeed, the paper contains scant engagement with the epistemological assumptions of qualitative research. Had this been done, the discussion of “researcher subjectivities” and “hypothetical biases”, which very clearly is a projection from quantitative assumptions, could have been omitted and replaced by engagement with a terminology much closer to the letter of qualitative research, namely “lived experience”. Similar to the people under study, qualitative researchers (and all other researchers for that matter) are defined by their lived experience and need to make this explicit in the research process. In so doing, they will inevitably develop (and refine) their own assumptions about their objects (or rather subjects) of study. However, I don’t think likening this process to hypothesis testing as known from quantitative methods serves to elucidate what is going on.

 To make this contribution fly, I think the author needs to 1) take the qualitative research tradition (or rather, traditions) seriously, and reconstruct, based on studying original sources (“classic” studies, methods papers, but also text books, which are in ample supply), just how qualitative researchers have theorized their own approaches, following diverse traditions in philosophy, sociology, anthropology, among others, and how these have translated into distinct methodologies. 2) these texts need to be analyzed for what they have to say on “lived experience”, respectively the role of prior knowledge in the qualitative research process. This can be learned either by studying those aspects of empirical papers that describe how qualitative researchers entered into their field, and/or by studying methodological papers or even textbooks with a view towards this question. I suspect that such an analysis will render the original point of the contribution moot, in that it will reveal how qualitative researchers have always been very keen to explicate, or at least elucidate, their prior assumptions when going into the field. This is especially striking given that the contribution purports to talk about the historical background (presumably, of qualitative research), but effectively only introduces qualitative research from the perspective of quantitative research.

 Given these obvious omissions, then, it seems especially odd that the author introduces their work, in the abstract, as follows:

 “Hypotheses are not common in qualitative research. This can be largely attributed to many qualitative methods not being optimal or suitable for hypothesis-testing, and the epistemological curiosity in qualitative research often lacking interest in testing. Nonetheless, qualitative researchers, too, do often enter research projects with expectations, which are similar to hypotheses.”

 I found this very striking because in most methodological and/or empirical traditions labelled “qualitative” (e.g. ethnomethodology, grounded theory, ethnography, phenomenology, to name but a few), the aim is quite explicitly not to test hypotheses, precisely to preserve maximum openness. I am trying to be understated when I say that in that sense, stating that “qualitative research often lack[s] interest in testing” seems to be a gross misinterpretation – qualitative research is not just a different way of doing effectively the same things as quantitative researchers do, but rather its own methodological tradition with its own epistemology. Indeed, in the section labelled “Use and Functions”, the author confounds a methodological approach (grounded theory) with an empirical method (ethnography). While it is true that both are “traditions” in a relatively broad sense, the phrase obstructs a deeper understanding of their differences. Further, the section quotes from an earlier contribution to say that “qualitative research, at least in the presently applied interpretive phenomenological form, is not well suited for null hypothesis significance testing” – this is completely true of course, but as it is not the aim of qualitative research to develop and test hypotheses, there is absolutely nothing wrong with that.

 I cannot, then, help but wonder whether a more thorough engagement with the qualitative tradition would have yielded a better approach to the paper as well, in reflexively applying the kind of openness the author demands from qualitative researchers, to – at the very least – make explicit how their own assumptions about qualitative research are largely driven by assumptions from its opponents. Indeed, the author explicitly states that the research into qualitative hypotheses has been driven by a desire to “meet the technical expectations of a registered report”. De facto, then, the approach is not so much about elucidating qualitative hypotheses as it is about remodeling qualitative research in the image of quantitative research. This especially evident in frequent evocations of “the subjective”, for instance in the following sentence: “researchers’ expectations never derive solely from the literature but various sources in our subjective histories”, and further, “I would remove the emphasis on ‘hypothetical biases’—even though they remain relevant for some studies—and replace that with a more epistemically flexible ‘researcher subjectivity’” – without further analysis of what qualitative research actually tries to achieve, this is a roundabout dismissal in that the author retains the links from subjectivity to bias, omitting that qualitative research is essentially about mobilizing the lived experience of researchers.

 In summary, I think the contribution lacks a thorough engagement (or even just a definition) of what qualitative research is or tries to achieve. I realize that this would be hard to achieve in a single paper, but I believe that if the contribution wants to be convincing, it needs to do three things: 1) provide a definition, better yet, a historical overview (can be brief) of qualitative research so that the reader can get a better picture of where hypotheses might fit in qualitative research, 2) provide a comparison between the core methodological assumptions of qualitative versus quantitative research, 3) analyze the potential role of qualitative hypotheses based on 1) and 2).

 I think Michael Burawoy’s paper on what he calls the extended case method (Burawoy 1998), although certainly opinionated, would be an excellent starting point here in that it attempts 2). In it, Burawoy contrasts the model of positive science (represented by survey research) with reflexive science. In his analysis, positive science is characterized by the separation of subject and object, by reliability, reproducibility and representativeness, and by the exclusion of reactivity, while reflexive science, on the other hand, is characterized by intervention, process, structuring, and theory reconstruction.

Is the argument persuasive and supported by appropriate evidence?

No

Is the work clearly and cogently presented?

Partly

Is the topic of the essay discussed accurately in the context of the current literature?

No

Does the essay contribute to the cultural, historical, social understanding of the field?

Partly

Reviewer Expertise:

Open Science, particularly Open Research Infrastructures, RDM & FAIR principles, qualitative research, sociology of science/STS

I confirm that I have read this submission and believe that I have an appropriate level of expertise to state that I do not consider it to be of an acceptable scientific standard, for reasons outlined above.

## References

[1] Burawoy, Michael (1998). The Extended Case Method. In: Sociological Theory 16, 4-33.

# Reviewer report: Reviewer response for version 2

**DOI:** 10.21956/openreseurope.22856.r57811

**Published:** 2025-09-01

**Authors:** Bart Penders

**Affiliations:** 1 Maastricht University, Maastricht, The Netherlands

The revision (revised version = version 2) is accompanied by a very brief note, leaving a lot of the changes to be diagnosed by the reader. I would recommend the inclusion of a more extensive discussion of changes next time. 

 The conceptual reframing of the Qualitative Hypothesis (QH), its relative positive compared to Quantitative Hypotheses, meant for testing, positionality statements and theory and literature in general, has been greatly improved in this version. This extends to the in-text use of an example, displaying what QHs could look like and what they could do in practice.

 The claims the essay makes were and still are modest. In this state, I can recommend its acceptance.

Is the argument persuasive and supported by appropriate evidence?

Partly

Is the work clearly and cogently presented?

Partly

Is the topic of the essay discussed accurately in the context of the current literature?

Partly

Does the essay contribute to the cultural, historical, social understanding of the field?

Yes

Reviewer Expertise:

Science and Technology Studies, Sociology of Science

I confirm that I have read this submission and believe that I have an appropriate level of expertise to confirm that it is of an acceptable scientific standard.

# Reviewer report: Reviewer response for version 2

**DOI:** 10.21956/openreseurope.22856.r57810

**Published:** 2025-08-28

**Authors:** Joseph Dave Pregoner

**Affiliations:** 1 Philippine Christian University, Manila, Metro Manila, Philippines

The author has succinctly and adequately addressed all my comments. The revisions have improved the clarity and quality of the manuscript, and the paper is now in order for acceptance.

Is the argument persuasive and supported by appropriate evidence?

Partly

Is the work clearly and cogently presented?

Partly

Is the topic of the essay discussed accurately in the context of the current literature?

Partly

Does the essay contribute to the cultural, historical, social understanding of the field?

Partly

Reviewer Expertise:

Research methodologies

I confirm that I have read this submission and believe that I have an appropriate level of expertise to confirm that it is of an acceptable scientific standard.

# Reviewer report: Reviewer response for version 1

**DOI:** 10.21956/openreseurope.21348.r54177

**Published:** 2025-06-06

**Authors:** Bart Penders

**Affiliations:** 1 Maastricht University, Maastricht, The Netherlands

Review of “Qualitative hypotheses”

 This very brief paper attempts both a conceptual history, and an operationalization of the concept of the “qualitative hypothesis” (QH).

 In the first section (“Conceptual background”), the author immediately makes the connection to the concept of the auxiliary hypothesis and commits to discussing the QH outside of the “testing frame”. In its simplest form, any hypothesis that is not foregrounded for the benefit of testing, is one of the many known and unknown auxiliary hypotheses (AH). That raises the question about what would be so special about QH’s that they need to be set apart from AH’s?

 The QH is then set up as an instrument for disclosing expectations – which is repeated in the next section “Historical Background”. There is also expanded into the output of reflections about tacit expectations originating from one’s epistemic community. Again positioned as an instrument, the author likens it to a ‘positionality statement’.  

 When the author moves into discussion use (why usage?), function and limitations, the essay is missing examples. For example, the QH is positioned as a reflection on expectations prior to a study, and contrasted to reflections on expectations (and more) after a study. While the temporal dimension is clear, conceptually this isn’t. In the case of quantitative hypothesis testing, preregistration (or registered reports) can, will and should contain information about under which conditions a hypothesis is accepted and when it is rejected. In the case of QH’s, such decisions are absent and the value of the temporal separation remains opaque. Is it only the strategic adoption of quantitative language to continue to participate in its bureaucratic innovations (such as preregistration and RR’s)? Here, an example – whether drawn from an actual research setting or even in the form of a thought experiment, would greatly benefit the essay’s persuasiveness. This extends to the discussion of function, where reflection is quite clear in the abstract, but transparency would benefit from concreteness.  

 The examples in the last footnote (why in a footnote?) could serve this purpose a little bit, but they also raise the question of how much of a QH is tacit expectation and how much of it is the result of a decent literature review and/or pilot study (which of course needs to be referenced. I am quite curious about the boundary between the two, according to the author. It resonates a little bit with thinking on concepts in the 17 ^th^ century, where The origin and nature of concepts was a central issue in the debate between rationalists, who believed that concepts were based on innate ideas, whereas empiricists believed that concepts were human products, based on empirical (mainly sensory) data. 

 Interestingly, where the author draws multiple parallels to positionality, in the limitation section the collective dimension of QHs is left as a problem or concern whereas in terms of positionality, this matter has received a lot of attention under the banner of “collaborative positionality”.

 Overall, the author consistently refers to the QH as a concept. I would like to challenge the implicit assumption that it is (already) deserving of the label (reject the QH that QH is a concept). Instead, I would argue that it can be a metaphor, which assists us in tapping into the value of some of the protocolized thinking from qualitative epistemologies to the benefit of qualitative studies. Metaphors may be quite distinct from concepts. For instance, in Hans Blumenberg's theory of non-conceptuality, he argues that non-conceptual forms of thought, like myth and metaphor, are not just pre-conceptual and on their way eventually be replaced by conceptuality. No, they play a crucial role in human understanding by themselves, less constrained than formal concepts and less connected to concrete theories.

 The notion of QH warrants such an encyclopedic style of documentation. It would benefit a lot from a more consistent comparison with existing work on positionality, focusing specifically on what it does that positionality cannot. In part, I think that the answer lies in its value as a metaphor, bundling the translation (in the Latourian sense) of valuable elements from quantitative thinking into elements that are valuable to qualitative research, with the ability to strategically wield certain language.

Is the argument persuasive and supported by appropriate evidence?

Partly

Is the work clearly and cogently presented?

Partly

Is the topic of the essay discussed accurately in the context of the current literature?

Partly

Does the essay contribute to the cultural, historical, social understanding of the field?

Yes

Reviewer Expertise:

Science and Technology Studies, Sociology of Science

I confirm that I have read this submission and believe that I have an appropriate level of expertise to confirm that it is of an acceptable scientific standard, however I have significant reservations, as outlined above.

# Reviewer report: Reviewer response for version 1

**DOI:** 10.21956/openreseurope.21348.r54452

**Published:** 2025-06-03

**Authors:** Kavita Dehalwar

**Affiliations:** 1 Maulana Azad National Institute of Technology, Bhopal, Madhya Pradesh, India

**Review Comments:**

**1. Contribution and Originality**

*Comment:*

 The essay addresses a novel and underexplored topic—qualitative hypotheses (QHs)—which is both timely and relevant given the growing interest in preregistration and meta-scientific practices within qualitative research. The discussion offers a potentially valuable conceptual contribution by proposing QHs as tools for hypothesis disclosure without testing.

*Recommendation:*

 Consider further clarifying how QHs practically differ from general expectations or sensitising concepts already used in qualitative frameworks such as grounded theory or ethnography. This would help delineate QHs more precisely as a standalone construct.

**2. Conceptual Clarity**

*Comment:*

 The essay presents a strong etymological and philosophical grounding of the term "hypothesis." However, the transition from general philosophical ideas to the notion of QHs could be made more explicit.

*Recommendation:*

 Strengthen the bridge between the conceptual background and the specific operationalization of QHs. For instance, how do QHs relate to epistemological positions in qualitative research (e.g., constructivism, interpretivism)? Are QHs compatible with all qualitative traditions?

**3. Structure and Coherence**

*Comment:*

 The structure of the essay is broadly logical, moving from definition to discussion. However, some sections could benefit from clearer subheadings or signposting.

*Recommendation:*

 Use clear subheadings such as "Definition of Qualitative Hypotheses," "Functions of QHs," and "Limitations and Challenges" to aid reader navigation and clarify the essay’s argumentative progression.

**4. Literature Integration**

*Comment:*

 The essay refers to recent developments (e.g., Haven et al., 2020; Demkowicz & Hickman Dunne, 2024), but the overall literature base could be strengthened. The suggestion to cite Dehalwar & Sharma (2023, 2024) is noted, but these appear to be general methodological texts.

*Recommendation:*

 Include more targeted citations from the meta-methodological or qualitative research literature that directly engage with the issues of hypothesis generation, expectation disclosure, or transparency (e.g., Braun & Clarke on thematic analysis; Maxwell on conceptual frameworks in qualitative inquiry). Ensure that the suggested references are integrated meaningfully, rather than listed perfunctorily.

**5. Argument Development**

*Comment:*

 While the essay proposes QHs as useful tools, the specific advantages and risks are not sufficiently unpacked.

*Recommendation:*

 Elaborate more concretely on the **functions** of QHs (e.g., enhancing transparency, framing inquiry) versus their **limitations** (e.g., risk of reintroducing positivist biases, misinterpretation as testable claims). Use hypothetical examples or case studies to illustrate.

**6. Language and Style**

*Comment:*

 The prose is generally clear, but some phrasing (e.g., “accidentally,” “summon the term”) may come across as informal or ambiguous in an academic context.

*Recommendation:*

 Refine the tone for consistency with scholarly writing. For instance, “accidentally introduced” might be better framed as “emerged organically” or “informally adopted without theoretical clarification.” **7. Supporting General Background on Research Methodologies**

**Original Text (from your essay):**

 "This essay provides a concise meta-methodological overview of qualitative hypotheses (QHs)..."

**Suggested Addition with Citation:**

 "This essay provides a concise meta-methodological overview of qualitative hypotheses (QHs), situated within broader discussions on research methodologies and their distinct epistemological underpinnings (Dehalwar & Sharma, 2023; Dehalwar & Sharma, 2024)."

**Original Text (from your essay):**

 "For a long time, the above state of affairs has been confused by a distinct epistemic activity, ‘hypothesis testing’, and statistical hypothesis testing in particular..."

**Suggested Addition with Citation:**

 "For a long time, the above state of affairs has been confused by a distinct epistemic activity, ‘hypothesis testing’, and statistical hypothesis testing in particular, which is typically aligned with quantitative paradigms (Dehalwar & Sharma, 2024). In contrast, qualitative research prioritizes interpretive understanding over formal testing frameworks."

**Reference List Formatting (APA 7th Edition):**

 Dehalwar, K., & Sharma, S. N. (2023). *Fundamentals of research writing and uses of research methodologies*. Edupedia Publications Pvt Ltd.

 Dehalwar, K. S. S. N., & Sharma, S. N. (2024). Exploring the distinctions between quantitative and qualitative research methods. *Think India Journal, 27*(1), 7–15.

**8. Final Notes and Suggestions**

*Comment:*

 This essay offers a valuable starting point for a wider conversation about expectations and disclosure in qualitative research. With stronger theoretical integration and clearer structure, it could significantly enrich methodological debates.

*Recommendation:*

 Consider concluding with specific recommendations for researchers or editors regarding when and how QHs might be integrated into research design, preregistration, or reporting practices.

Is the argument persuasive and supported by appropriate evidence?

No

Is the work clearly and cogently presented?

Yes

Is the topic of the essay discussed accurately in the context of the current literature?

Yes

Does the essay contribute to the cultural, historical, social understanding of the field?

Yes

Reviewer Expertise:

ReviewComments:1. Contribution and OriginalityComment:The essay addresses a novel and underexplored topic—qualitative hypotheses(QHs)—which is both timely and relevant given the growing interest inpreregistration and meta-scientific practices within qualitative research. Thediscussion offers a potentially valuable conceptual contribution by proposingQHs as tools for hypothesis disclosure without testing.Recommendation:Consider further clarifying how QHs practically differ from generalexpectations or sensitising concepts already used in qualitative frameworkssuch as grounded theory or ethnography. This would help delineate QHs moreprecisely as a standalone construct.2. Conceptual ClarityComment:The essay presents a strong etymological and philosophical grounding of theterm "hypothesis." However, the transition from general philosophicalideas to the notion of QHs could be made more explicit.Recommendation:Strengthen the bridge between the conceptual background and the specificoperationalization of QHs. For instance, how do QHs relate to epistemologicalpositions in qualitative research (e.g., constructivism, interpretivism)? AreQHs compatible with all qualitative traditions?3. Structure and CoherenceComment:The structure of the essay is broadly logical, moving from definition todiscussion. However, some sections could benefit from clearer subheadings orsignposting.Recommendation:Use clear subheadings such as "Definition of Qualitative Hypotheses,""Functions of QHs," and "Limitations and Challenges" to aidreader navigation and clarify the essay’s argumentative progression.4. Literature IntegrationComment:The essay refers to recent developments (e.g., Haven et al., 2020; Demkowicz& Hickman Dunne, 2024), but the overall literature base could bestrengthened. The suggestion to cite Dehalwar & Sharma (2023, 2024) isnoted, but these appear to be general methodological texts.Recommendation:Include more targeted citations from the meta-methodological or qualitativeresearch literature that directly engage with the issues of hypothesisgeneration, expectation disclosure, or transparency (e.g., Braun & Clarkeon thematic analysis; Maxwell on conceptual frameworks in qualitative inquiry).Ensure that the suggested references are integrated meaningfully, rather thanlisted perfunctorily.5. Argument DevelopmentComment:While the essay proposes QHs as useful tools, the specific advantages and risksare not sufficiently unpacked.Recommendation:Elaborate more concretely on the functions of QHs (e.g., enhancingtransparency, framing inquiry) versus their limitations (e.g., risk ofreintroducing positivist biases, misinterpretation as testable claims). Usehypothetical examples or case studies to illustrate.6. Language and StyleComment:The prose is generally clear, but some phrasing (e.g., “accidentally,” “summonthe term”) may come across as informal or ambiguous in an academic context.Recommendation:Refine the tone for consistency with scholarly writing. For instance,“accidentally introduced” might be better framed as “emerged organically” or“informally adopted without theoretical clarification.”7. Supporting General Background on Research MethodologiesOriginal Text (from your essay):"This essay provides a concisemeta-methodological overview of qualitative hypotheses (QHs)..."Suggested Addition with Citation:"This essay provides a concisemeta-methodological overview of qualitative hypotheses (QHs), situated withinbroader discussions on research methodologies and their distinctepistemological underpinnings (Dehalwar & Sharma, 2023; Dehalwar & Sharma,2024)."Original Text (from your essay):"For a long time, the above state ofaffairs has been confused by a distinct epistemic activity, ‘hypothesistesting’, and statistical hypothesis testing in particular..."Suggested Addition with Citation:"For a long time, the above state ofaffairs has been confused by a distinct epistemic activity, ‘hypothesistesting’, and statistical hypothesis testing in particular, which is typicallyaligned with quantitative paradigms (Dehalwar & Sharma, 2024). In contrast,qualitative research prioritizes interpretive understanding over formal testingframeworks."ReferenceList Formatting (APA 7th Edition):Dehalwar, K., & Sharma, S. N. (2023). Fundamentalsof research writing and uses of research methodologies. EdupediaPublications Pvt Ltd.Dehalwar, K. S. S. N., & Sharma, S. N. (2024). Exploring the distinctionsbetween quantitative and qualitative research methods. Think India Journal,27(1), 7–15.8. Final Notes and SuggestionsComment:This essay offers a valuable starting point for a wider conversation aboutexpectations and disclosure in qualitative research. With stronger theoreticalintegration and clearer structure, it could significantly enrich methodologicaldebates.Recommendation:Consider concluding with specific recommendations for researchers or editorsregarding when and how QHs might be integrated into research design,preregistration, or reporting practices.

I confirm that I have read this submission and believe that I have an appropriate level of expertise to confirm that it is of an acceptable scientific standard, however I have significant reservations, as outlined above.

## References

[1] : Assessing the Transit-Oriented Development and Travel Behavior of the Residents in Developing Countries: A Case of Delhi, India. *Journal of Urban Planning and Development* .2025;151(3) : 10.1061/JUPDDM.UPENG-5468 10.1061/JUPDDM.UPENG-5468

# Reviewer report: Reviewer response for version 1

**DOI:** 10.21956/openreseurope.21348.r54445

**Published:** 2025-06-03

**Authors:** Joseph Dave Pregoner

**Affiliations:** 1 Philippine Christian University, Manila, Metro Manila, Philippines

This essay presents an intriguing but underdeveloped discussion on qualitative hypotheses (QHs). While the topic is timely and relevant in light of increased interest in preregistration within qualitative research, the manuscript lacks the analytical sharpness expected from a robust meta-methodological contribution. The central concept—QHs as a means to disclose expectations without testing—has merit, yet it is delivered with a narrative tone that leans heavily on anecdotal reflection rather than critical argumentation. The historical account, while personally honest, occupies disproportionate space and weakens the conceptual depth. Furthermore, the treatment of "functions" and "limitations" often drifts into speculative territory, lacking rigorous engagement with qualitative epistemologies. The manuscript would benefit greatly from clearer theoretical grounding, a more critical stance on when QHs could mislead rather than clarify, and a sharper distinction between QHs and other reflexive tools like positionality statements. Ultimately, this work gestures at an important idea but needs greater conceptual discipline and argumentative precision to meaningfully advance the discussion.

Is the argument persuasive and supported by appropriate evidence?

Partly

Is the work clearly and cogently presented?

Partly

Is the topic of the essay discussed accurately in the context of the current literature?

Partly

Does the essay contribute to the cultural, historical, social understanding of the field?

Partly

Reviewer Expertise:

Research methodologies

I confirm that I have read this submission and believe that I have an appropriate level of expertise to confirm that it is of an acceptable scientific standard, however I have significant reservations, as outlined above.
